# From prevention of pin-tract infection to treatment of osteomyelitis during paediatric external fixation

**DOI:** 10.1007/s11832-016-0787-8

**Published:** 2016-11-15

**Authors:** Dimitri Ceroni, Catherine Grumetz, Odile Desvachez, Sophie Pusateri, Pierre Dunand, Eleftheria Samara

**Affiliations:** 1 Division of Paediatric Orthopaedics, University Hospitals of Geneva, Geneva, Switzerland; 2 Ambulatory Nursing Care Consultation for Paediatric External Fixators, University Hospitals of Geneva, Geneva, Switzerland

**Keywords:** Pin, Half-pin, Wire, Tract, Infection, Instability

## Abstract

Pin-tract infection (PTI) is the most commonly expected problem, or even an almost inevitable complication, when using external fixation. Left unteated, PTI will progress unavoidably, lead to mechanical pin loosening, and ultimately cause instability of the external fixator pin–bone construct. Thus, PTI remains a clinical challenge, specifically in cases of limb lengthening or deformity correction. Standardised pin site protocols which encompass an understanding of external fixator biomechanics and meticulous surgical technique during pin and wire insertion, postoperative pin site care and pin removal could limit the incidence of major infections and treatment failures. Here we discuss concepts regarding the epidemiology, physiopathology and microbiology of PTI in paediatric populations, as well as the clinical presentations, diagnosis, classification and treatment of these infections.

## Introduction

External fixation has become a key tool in the orthopaedic surgeon’s modern armamentarium, being used both in traumatology and reconstructive surgery. The key advantage of this method is that the implant assembly—called the external fixator device—is located outside the body and is connected to the bone via transcutaneous pins or wires. External fixators were first applied to treat comminuted fractures, open fractures and bone loss, but its applications broadened in subsequent years, notably after Ilizarov’s development of distraction osteogenesis [1, 2], and external fixation is currently also used to correct congenital and acquired deformities, mobilise stiff joints and heal infected nonunions. However, external fixation is associated with high rates of morbidity, especially when a prolonged application is necessary [3], with inflammation and subsequent infection at the metal–skin interface—more commonly known as pin-tract infection (PTI)—being one of the most common problems encountered, with reported rates ranging from 1 to 100% [4–15]. This great discrepancy in reported incidences of PTI is partly due to the lack of a uniform definition and classification system for the determination and quantification of this type of infection. At the present time, PTI infection is broadly defined as the signs and symptoms of infection around pins or wires that require the administration of an antibiotic, pin or wire removal, or even surgical debridement. In this current opinion review, we discuss the concepts regarding the epidemiology, physiopathology and microbiology of PTI in paediatric populations, as well as the clinical presentations, diagnosis, classification and treatment of this type of infection.

## Pathogenesis

There is currently a lack of clear evidence and consensus on the pathogenesis of PTI, and many apparently contradictory hypotheses have been described. Numerous authors [3, 4, 16, 17] have reported that pin tract problems always develop from the outside to the inside, a hypothesis based on the belief that such problems start with a soft tissue inflammation that leads to soft tissue infection and finally to bone infection. According to this hypothesis, PTI can then spread through the continuity of the bone—despite the absence of any mechanical pin loosening—and result in colonisation of the medullary canal. In this case, the stability of the internal fixator is impaired, and infection may persist even after the removal of the wire or pin. This theory associates PTI with pin–skin motion, the amount of soft tissue between skin and bone and the diameter of the pin used [3]. Clasper et al. [16] also incriminated fluid accumulation around the pin–bone interface as a cause of PTI.

This description of the development of PTI has been disputed by other authors [14, 18] who believe that it is a pathophysiological misconception to consider that pin loosening results from PTI. According to their theory, it is the instability of the external fixator pin–bone construct that leads to pin loosening and infection and, consequently, it is pin loosening that is the initiating event which ultimately leads to pin tract sepsis. In this hypothesis, the external fixator construct appears to be vital to the prevention of pin site complications since excessive movement at the fixator pin–bone interface leads to pin site irritation and infection. There is probably an ongoing race between the gradually increasing load capacity of the healing bone and the potential for failure at the bone–pin interface. An unstable fixator will create a mechanically unfavourable environment for optimal bone healing and lead to deleterious instability at the fixator pin–bone interface, thereby producing pin tract irritation and then infection [14, 18].

### Bacterial colonisation of pin and biofilm production

Pin or wire colonisation by bacteria starts during surgery or in the early postoperative period and has been described as occurring in steps [19, 20]. Immediately after insertion of the wire or pin, plasma proteins rapidly coat the surface of the fixation pin implant [21]. The current belief is that the initial interaction between bacteria and the adsorbed proteins is probably non-specific, occurring through a combination of Van der Waals, gravitational and Coulombic forces [22]. Membrane proteins and polysaccharides subsequently allow the bacteria to bind firmly to the proteins on the device surfaces. Finally, certain bacterial species secrete a protective exopolysaccharide layer—the biofilm—which renders them resistant to antibiotics [23, 24]. Biofilm-related bacterial infections are recognised as being exceedingly difficult to treat with conventional systemic antibiotic therapies [24], thus validating the promising strategy of seeking to inhibit bacterial adhesion prior to biofilm formation.

*Staphylococcus epidermidis*, *S. aureus* and *Escherichia coli* are the three most common infective agents of external fixation constructs [25]. *Staphylococci* are recognised as the most frequent causes of biofilm-associated infections. Their exceptional status among biofilm-associated pathogens is due to the fact that staphylococci are frequent commensal bacteria on human skin and mucus. They are thus among the most likely germs to infect any medical device that penetrates these surfaces, such as those being inserted during surgery [26]. Recent advances in our knowledge of staphylococcal molecular biology have provided a more detailed insight into biofilm formation by these opportunistic pathogens. A series of surface proteins mediate the initial attachment to host matrix proteins, and then the expression of a cationic glucosamine-based exopolysaccharide aggregates the bacterial cells. Similarly, and like many other Gram-negative microorganisms, *E. coli* also has the capacity to form biofilm structures in vivo and in vitro [27, 28].

## PTI classification

Troublingly, classification of PTI varies throughout the literature, with some studies considering such subjective symptoms as pain, whereas others use clinical judgments of infection, radiological signs or microbiological diagnosis of infection. Unfortunately, any classifications are closely correlated to responses to treatment, indicating the retrospective nature of their usage. Thus, a clear systemic methodology for describing PTI is still lacking and, above all, there exists no validated grading system to evaluate the severity of this problem. Many PTI classifications are overly subjective, with varying inter-rater reliability when grading pain, the extent of erythema, tenderness and swelling at a pin site suspected of an infection. Additionally, a grading system which includes pain as a criterion may vary significantly based on cultural or social backgrounds [29].

Based on clinical symptoms, such as erythema and pain, Clint et al. [30] described a simple approach that classifies pin sites as “good”, “bad” or “ugly”. Similarly, Santy et al. [31] established criteria for describing pin sites as “calm”, “irritated” or “infected” that take into account patients’ and clinicians’ observations. Ward et al. [32] categorised minor PTI as the presence of prolonged discharge, swelling and crusting, all clinical features that may be controlled using oral antibiotics, whereas major infection requires surgical drainage and removal of pins. Paley et al. classified PTI gradually, starting with soft tissue inflammation, then soft tissue infection and finally bone infection [3]. Other authors have graded the severity of the pin infection according to the presence of purulent discharge, skin erythema and radiological evidence of wire or half-pin loosening [33]. Checketts et al. [34] reported a classification system for PTI consisting of three grades of minor infections and three grades of major infections. This system considers clinical features and radiological evidence of osteolysis, with the significant difference between the two groups being that the external fixation has to be abandoned in major infections (Table 1). A last classification, described by Saleh and Scott, tries to grade the PTI according to therapeutic response to different treatments; however, as this system is retrospective in nature, it cannot be used as a predictive tool [35].

**Table 1:** Checketts–Otterburn grading system for level of pin site infection

Grade	Appearance	Treatment
	Minor infection	
1	Slight redness, little discharge	Improved pin site care
2	Redness of skin, discharge, pain and tenderness in the soft tissue	Improved pin site care, oral antibiotics
3	Grade 2 but not improved with antibiotics	Affected pin or pins resited and external fixation continued
	Major infection	
4	Severe soft tissue infection involving several pins, sometimes with associated loosening of the pin	External fixation must be abandoned
5	Grade 4 but also involvement of the bone; also visible in radiographs	External fixation must be abandoned
6	The infection occurs after fixation removal. The pin track heals initially but will break down and discharge at intervals Radiograph shows new bone formation and sometimes Sequestrum	Curettage of the pin track

## How to prevent PTI?

It is widely agreed that any strategy for reducing PTI begins in the operating theatre [17], but it could be legitimately suggested that PTI prevention should start even earlier, i.e. during the surgical planning step. Choosing the correct external fixator is probably a crucial issue, especially for limb lengthening or deformity correction. Indeed, incidences of PTI during limb lengthening and lower-limb reconstruction are elevated [4, 18]. The high incidence of PTI reported in limb reconstruction surgery may be related to the long periods of time spent in the external fixator and high demands placed on the bone–pin interface during either bone transport or deformity correction [18]. The primary goal of planning should be to ensure a stable bone–pin interface that will withstand the stresses transferred during the reconstructive period [14, 36] and, therefore, this criterion should determine the choice of the appropriate fixator for the planned surgery. Parameswaran et al. [14] demonstrated that the type of fixator had an effect on the incidence of PTI, with monolateral and hybrid fixators having a much higher incidence of PTI than ring fixators. In addition, Antoci et al. [14.] demonstrated that the incidence of PTI was higher with half-pin external fixators than with hybrid fixators using fine wires in addition to half-pins [4]. As a general rule, it seems that a half-pin site is more prone to PTI than a fine-wire site [4]. Interestingly, the Russian school demonstrated that elastic stable intramedullary nailing could stimulate new endosteal and periosteal bone formation and thus decrease the high incidence of PTI reported in limb reconstruction surgery due to the long periods spent in the external fixator [37].

## Technical notes for surgery

Many authors advocate that great efforts should be made to ensure that not only pin and wire insertion is as atraumatic as possible for the skin, but also for soft tissue and bone, thereby minimising iatrogenic damage to these structures. Thus, the location or placement of the pin must be considered carefully. Skin incisions should only be as large as the diameter of the pin [18], and these incisions should be made with care in order to avoid tension on the skin. Immediate subcutaneous bone surfaces are preferable, since pins located in areas with considerable soft tissue, tendons and tendon sheaths are at the greatest risk of infection [38, 39]. Wires should not be drilled through to soft tissue, but rather pushed into the near cortex, then drilled through the bone and finally advanced through the opposite soft tissue by tapping with a mallet [40]. Any muscle compartment traversed during the placement pins or wires should be placed under stretch [38, 39] in order to prevent transfixing muscles in a shortened position [38, 39]. Heat generation must be avoided during pin or wire insertion, as this could lead to thermal necrosis of the surrounding bone, ring sequestra and pin loosening. Thus, the anterior tibial crest should be avoided at all cost, as drilling through the thick cortical bone can generate excessive heat [18, 38]. It is thus advisable to drill using continuous cold saline irrigation to ensure proper pin cooling [17]. For half-pin placement, pre-drilling should always be performed, even when using self-drilling pins [38]. After drilling, the pilot hole must be irrigated to remove any bone swarf that might act as sequestra and prevent optimal bone–pin fixation [17, 18]. Finally, many authors follow Davies’ recommendations and, as far as possible, use a no-touch technique when inserting half-pins [17, 18]. To ensure a no-touch technique for inserting wires, chlorhexidine or alcoholic iodine-soaked swabs are used to handle and manipulate wire placement. The immediate use of pressure dressings and the removal of any blood from the skin, especially around the pin site, also lessen the proliferation of bacteria within a haematoma and minimise pin–skin motion.

## New pins with bactericidal effects

A number of technical advances have been made to reduce the risk of PTI while maintaining pin stability. Clinicians have attempted numerous methods to solve the problem of PTI [41], including applying external electromagnetic fields, using alloys to manufacture pins or coating the pin with antibiotics or chemical substances such as hydroxyapatite [36, 42–49], hydroxyapatite with chlorhexidine [47, 50], silver nanoparticles [51–56], chlorhexidine, zinc or titanium oxide [57] and micron-thin sol–gel films [58].

Hydroxyapatite-coated pins show improved fixation strength, with extraction torque forces that are higher than the initial torque forces and 90-fold higher than those of standard uncoated pins [59]. This improved fixation translates into lower rates of osteolysis and subsequently to lower incidences of pin loosening and lower pin site infection than uncoated pins [36, 42–44, 46, 49]. Hydroxyapatite–chlorhexidine-coated pins exhibit the dual benefits of enhanced bone stability through bonding to the pin (due to hydroxyapatite) and localised release of chlorhexidine. Silver-coated pins decrease bacterial colonisation [60] and result in fewer infections and PTI [53]. Unfortunately, silver-coated pins may induce cytotoxicity [61–63] as some authors have found significant amounts of silver in blood serum [60]. Diffused silver in the blood serum may act as a Trojan horse by entering cells and then releasing silver ions that damage intracellular function [63]. The bactericidal effect induced by nano-titanium dioxide (TiO_2_) exposed to ultraviolet radiation has been used successfully in many areas, such as the disinfection of water and textiles and in other cleaning processes [64–66]. When irradiated by near-ultraviolet light, nano-TiO_2_ shows strong bactericidal activity [65, 67]. One new, attractive perspective for combating PTI is the covalent bonding of antibiotics onto the surface of a titanium pin [4]. Other clinicians, such as Forster et al. [68] have assessed the potential of antibiotic polyurethane sleeves to inhibit bacterial colonisation on pins; based on their results, they concluded that the use of such sleeves should reduce the incidence of PTI.

## Care at the pin site

There is currently no universally accepted protocol for the optimal care of pin sites [40]. A myriad of protocols for pin site care has been described, with significant variations in nearly all aspects of care in terms of types of disinfection solutions, cleansing methods, dressing materials and, above all, the frequency of dressing changes [69]. Thus, pin site care protocols range from a nihilistic approach advocating no active pin care [66] to aggressive regimens involving twice-daily cleaning, dressing and oral antibiotics for the entire length of the treatment with an external fixator [14].

The appropriate time to start pin site care varies greatly in the literature, ranging from 24 h to 10 days after surgery [14, 17, 18, 38–40, 66, 70, 71]. Most of the time, the first fresh dressing is applied within 2 days of surgery, since gauzes are usually blood-soaked and occlusive crusts can appear around the pins. The frequency of pin site cleaning also differs, with authors suggesting cleaning twice daily [14, 72], once daily [39, 73], weekly [74] and, more rationally, “when required” [70]. In fact, the frequency of pin site cleaning should be correlated with the local status around the pin–tract site. If swelling, crusts or signs of an exudate are observed, then the frequency of pin site cleaning should be more regular (once every day or second day). Once the pin sites have healed and are clean and dry, the frequency of pin site cleaning can decrease, and it is recommended that dressings be replaced weekly.

Various cleaning solutions have been advocated in the literature, including soap and water, sterile water, normal saline, peroxide, polyvinylpyrrolidone-iodine, isopropyl alcohol, polyhexamethylene biguanide and chorhexidine aqueous or alcoholic solutions [14, 17, 18, 38–40, 70–73, 75]. It should be remembered that prolonged skin contact with strong antiseptic solutions may lead to dry skin, skin irritation or even a hypersensitive reaction [18]. Fortunately, such adverse effects can usually be resolved by substituting a strong antiseptic solution with a mild antiseptic soap and water. Once pin sites have healed and are clean, patients are allowed to shower, provided that the limb and its external fixator are carefully tested thereafter [18]. In accordance with the recommendations of most authors, we do not advise swimming, but swimming in a chlorinated pool may be permitted in specific cases if beneficial to bone healing.

There is also controversy over which kind of dressing to use after pin tract cleaning. Regardless of the dressings chosen, their main purpose is to keep pin sites clean and dry and to absorb any blood and exudates [70]. Many authors consider that pins should be dressed with sterile gauze in the presence of exudates, but left uncovered in their absence [4, 40]. Others advocate impregnating gauzes with antiseptic solutions in order to decrease the rates of PTI, such as benzalkonium chloride antiseptic solution [14], polyurethane [74], polyhexamethylene biguanide [75] or an alcoholic solution [17]. Paley reported using antibiotic-soaked sponges over pin sites [3]. We do not recommend the use of betadine-soaked gauzes as they induce crust formation and probably induce the skin to stick to the pins. New gauzes have been developed to promote skin healing and decrease PTI. In the presence of abundant exudates, hydrofibre dressings are useful due to their absorptive capacity. Antimicrobial silver dressings have also gained popularity due to the increase in antibiotic-resistant pathogens [76–78]. These are also interesting for their capacity to reduce the microbial contamination of wounds from environmental sources [79, 80] and, above all, because these dressings may be left in place for up to 7 days [81].

How the gauze should be fixed in place is yet another topic for discussion. The Russian protocol suggests the use of bulky pressure dressings to restrict movement between the skin and pins [37]. Paley [3] also recommends minimising pin–skin motion by applying pressure to the skin, either by using gauze compressed by rubber or by using foam sponge cubes pushed down using plastic clips. In a similar approach, Hoffmann [82] recommended relieving the skin tension around pins in order to prevent infection.

## Treatment of pin tract infection

When planning to use external fixation, orthopaedic surgeons should expect many of their patients to develop a PTI, particularly when lengthening limbs or correcting deformities. The most common bacterial etiology for PTI is cocci-shaped Gram-positive bacteria (methicillin-sensitive* S. aureus*, *S. epidermidis* and * Streptococcus* species) [4, 12, 53]. Rare cases of PTI involving methicillin-resistant* S. aureus* have been observed, especially when chronic osteomyelitis is present. Gram-negative bacteria may also be responsible for PTI, and pathogens such as *E. coli*,* Pseudomonas aeruginosa*,* Klebsiella pneumoniae*,* Enterococcus faecalis*,* Serratia marcecens* and *Vibrio vulnificus* may develop at the pin site. In rare cases, a mixed flora has even been identified [4]. Thus, it would appear that pin site swabs are required before any antibiotic treatment is initiated.

The Checketts–Otterburn PTI classification is commonly used as a guide to decision-making because it provides valuable information regarding treatment [34]. This classification distinguishes between two groups of PTI—minor (grades 1–3) and major (grades 4–6)—with the main difference being that the external fixation pin has to be removed in cases of major infection (see Table 1). Although PTI is common, very few cases lead to major complications, and most PTI are mild and may therefore respond to increased local pin site care. It is thus legitimate and safe to start treatment of a grade 1 PTI by increasing the frequency of local cleaning and dressing changes; it is also advisable to use more absorbent dressings in cases with excessive exudate. Hydrofibres can absorb large amounts of wound fluids, including exudate with bacteria. This is then transformed into a soft gel which creates a moist environment to support the body’s healing process. Furthermore, silver-releasing dressings have been proven to be as effective as oral antibiotics for controlling PTI; they could thus constitute the first-line therapy [78]. For a grade 2 Checketts–Otterburn PTI, not only should pin site care be improved, but patients should be treated with a course of oral antibiotics. Swabbing of the infected pin site is advised before initiating 7–10 days of oral antibiotics aimed at *S. aureus*. If the PTI resolves within that 7- to 10-day course of antibiotic, the medication can be discontinued, and regular pin site care may be resumed. A patient with grade 3 Checketts–Otterburn PTI should be admitted to hospital for intravenous antibiotics, inpatient pin site care and limb elevation. If these grade 3 PTI do not respond adequately to treatment, the pins or wires involved should be removed and changed, but external fixation can continue.

Major PTI, i.e. grade 4–6 Checketts–Otterburn PTI, should be managed by removing the infected pins or wires and performing an adequate debridement of the pin tracts to remove all necrotic debris [18, 83]. In cases of osteomyelitic pin tracts with sizeable cavities following debridement, the cavities can either be treated by leaving antibiotic beads in the tracts [14] or by using absorbable calcium-sulphate pellets impregnated with antibiotic to back-fill those tracts [38]. However, it is essential to remember that pin or wire removal must not destabilise the frame construction, as this will result in increased movement at the fixator pin–bone interfaces of the remaining pins and wires, with the potential for further infection [14]. The generally acceptable, and most preferable strategy is to re-situate the septic pins and wires rather than simply removing and replacing them, noting that all of these actions should be done without the overall external fixation being abandoned [18].

## Conclusion

Pin tract infection is an almost inevitable complication when using external fixation. It remains a clinically challenging problem, especially in treatments involving limb lengthening or deformity correction. Standardised pin site protocols that encompass an understanding of external fixator biomechanics and meticulous surgical technique during pin and wire insertion, postoperative pin site care and removal could limit the incidence of major infections and treatment failures.
